# The Duration of the Effects of Repeated Widespread Badger Culling on Cattle Tuberculosis Following the Cessation of Culling

**DOI:** 10.1371/journal.pone.0009090

**Published:** 2010-02-10

**Authors:** Helen E. Jenkins, Rosie Woodroffe, Christl A. Donnelly

**Affiliations:** 1 Department of Infectious Disease Epidemiology, Imperial College London, London, United Kingdom; 2 Institute of Zoology, London, United Kingdom; St. Petersburg Pasteur Institute, Russian Federation

## Abstract

**Background:**

In the British Isles, control of cattle tuberculosis (TB) is hindered by persistent infection of wild badger (*Meles meles*) populations. A large-scale field trial—the Randomised Badger Culling Trial (RBCT)—previously showed that widespread badger culling produced modest reductions in cattle TB incidence during culling, which were offset by elevated TB risks for cattle on adjoining lands. Once culling was halted, beneficial effects inside culling areas increased, while detrimental effects on adjoining lands disappeared. However, a full assessment of the utility of badger culling requires information on the duration of culling effects.

**Methodology/Principal Findings:**

We monitored cattle TB incidence in and around RBCT areas after culling ended. We found that benefits inside culled areas declined over time, and were no longer detectable by three years post-culling. On adjoining lands, a trend suggesting beneficial effects immediately after the end of culling was insignificant, and disappeared after 18 months post-culling. From completion of the first cull to the loss of detectable effects (an average five-year culling period plus 2.5 years post-culling), cattle TB incidence was 28.7% lower (95% confidence interval [CI] 20.7 to 35.8% lower) inside ten 100 km^2^ culled areas than inside ten matched no-culling areas, and comparable (11.7% higher, 95% CI: 13.0% lower to 43.4% higher, p  =  0.39) on lands ≤2 km outside culled and no-culling areas. The financial costs of culling an idealized 150 km^2^ area would exceed the savings achieved through reduced cattle TB, by factors of 2 to 3.5.

**Conclusions/Significance:**

Our findings show that the reductions in cattle TB incidence achieved by repeated badger culling were not sustained in the long term after culling ended and did not offset the financial costs of culling. These results, combined with evaluation of alternative culling methods, suggest that badger culling is unlikely to contribute effectively to the control of cattle TB in Britain.

## Introduction

Public controversy surrounds efforts to control bovine tuberculosis (TB) in the British Isles. Although bovine TB's causative agent (*Mycobacterium bovis*) primarily affects cattle, other mammalian hosts can be infected, including humans [1] and a number of wildlife species [2]. In the British Isles, control of cattle TB has been hampered by transmission of infection from wild badgers (*Meles meles*), and various forms of badger culling have been implemented to try to reduce such transmission [3]. Despite these efforts, the incidence of cattle TB remains high in both Britain and Ireland [4], [5], with 2,738 confirmed herd breakdowns in Britain in 2008 [4] and national expenditure of over £100 million. This situation has provoked heated debate as cattle TB can profoundly affect farmers' livelihoods, yet culling of badgers – which are nationally protected in the UK by their own Act of Parliament (http://www.opsi.gov.uk/ACTS/acts1992/ukpga_19920051_en_1) – is unpopular with the general public [6].

In 1998, the UK government launched a large-scale field trial (the Randomised Badger Culling Trial, RBCT) to assess the potential contribution of badger culling to the control of cattle TB [7]. The incidence of cattle TB in and around 10 large (100 km^2^) areas subjected to annual badger culling was compared with that in and around 10 matched areas with no such culling. While culling was ongoing, it was associated with a modest reduction in the incidence of cattle TB inside culled areas; however this beneficial effect was almost cancelled out by an increase in cattle TB incidence on adjoining unculled land [8], [9]. These simultaneous beneficial and detrimental effects meant that, over the five-year culling period, the financial costs of conducting any form of culling far outweighed the savings achieved through reductions in the numbers of cattle herds experiencing TB breakdowns [8], [10].

In the two years after culling ended, however, greater benefits became apparent: the positive effects inside culled areas became more pronounced, while the detrimental effects on adjoining land were no longer apparent [11]. Nevertheless, at that time the numbers of breakdowns prevented during and after culling were still not sufficient to offset the financial costs of conducting the culls [11]. Informed by these findings, and considering other factors such as practicality and public acceptability, the Secretary of State for Environment decided against badger culling to control cattle TB in England (http://www.defra.gov.uk/news/2008/080707b.htm). However, the Welsh Assembly Government proposes to implement a badger cull using methods to identify culling areas, and to cull badgers, very similar to those used in the RBCT (http://www.wales.gov.uk/bovinetb; though it faces a legal challenge to this proposal http://www.badger.org.uk/_Attachments/Resources/326_S4.pdf). Culling is also being considered in Northern Ireland (http://www.dardni.gov.uk/tb-statement.pdf).

The cost-effectiveness of badger culling as a cattle TB control measure depends in part on the duration of the benefits it imparts. If the effects are long-lasting, then the long-term benefits (in terms of breakdowns prevented) might offset the medium-term costs (in terms of the financial costs of culling, as well as the additional breakdowns on adjoining land prompted by culling). Here, we use updated cattle TB incidence data from RBCT areas to determine the duration of the effects of repeated widespread badger culling on cattle TB following the cessation of culling.

## Methods

Data presented here come from RBCT areas subjected to proactive culling (widespread culling, repeated approximately annually) and from their matched no-culling controls. RBCT methods are described in detail in refs [9] and [10] but, in brief, thirty 100km^2^ RBCT areas, arranged as 10 “triplets”, were selected in areas of England with high cattle TB incidence. Triplet locations are provided in ref [9]. All trial areas within each triplet were surveyed for badger activity before being randomly assigned to treatments such that each treatment – proactive culling, no culling, or localised “reactive” culling (conducted in response to specific TB breakdowns in cattle herds) – was replicated 10 times, once within each triplet. Badgers were captured in cage traps and despatched by shooting with a pistol; capture protocols took careful account of badger welfare [12], [13] and despatch was deemed ‘humane’ by independent audit [14]. Initial culls for each proactive trial area were completed between December 1998 and December 2002. Proactive culls were repeated approximately annually until culling ended in October 2005.

Data on the incidence of confirmed cattle TB breakdowns were downloaded from Defra's VetNet database, for herds inside RBCT areas and on adjoining land up to 2 km outside RBCT areas. Following ref [11] (which presented analyses of data available in January 2008), we analysed incidence data from two periods. We defined the “during-trial” period as running from the end of the initial proactive cull in each triplet, to exactly one year after completion of the last cull in that triplet, when another annual cull would have been conducted had the proactive treatment been continued. We defined the “post-trial period” as running from the end of the during-trial period up to the most recent data download (7th July 2009). To examine temporal trends, we further divided the during-trial period into intervals between successive culls (e.g. third to fourth cull), and divided the post-trial period into six-month intervals.

As in previously published analyses [8], [9], [11], [15], we used log–linear Poisson regression to compare the numbers of confirmed breakdowns recorded in and around trial areas subjected to the proactive and no-culling treatments. The regression models adjusted for triplet, the log of the number of baseline herds at risk, and the log of the number of confirmed breakdowns recorded in a three year period before RBCT culling commenced. Where results were stratified by time, a triplet*time interaction term was also included in the model. We adjusted confidence intervals (CI) and p-values for any extra-Poisson overdispersion by using an adjustment factor (the square root of the model deviance divided by the degrees of freedom) in all cases where its value was greater than 1.

Following examination of effects by six-month interval in the post-trial period, we fitted a linear trend (on a log scale) to the effects inside trial areas, and tested this trend against the null hypothesis of no trend. Additionally, in the adjoining areas, we grouped together the first 18 months of the post-trial period and tested the effect in this time period against the null hypothesis of no effect. We used previously published methods [10], [11] to investigate whether the effect of culling varied with distance from the trial area boundary.

As in previously published analyses [10], [11], we extrapolated from our results to estimate the size of an idealised circular culling area that would need to be targeted to obtain an overall reduction in the incidence of confirmed breakdowns, with detrimental effects outside the targeted area offset by beneficial effects inside. These extrapolations covered the time period from completion of the first proactive cull until effects were no longer detectable.

We calculated the financial costs and benefits of culling, using estimates of the costs of culling, and the benefits of preventing a breakdown, from ref [16]. The benefits included the prevention of both direct and indirect costs associated with: the loss of slaughtered cattle; movement restrictions; isolation; spread to other herds; as well as cattle testing (of the affected herd until the breakdown is cleared, of contiguous herds and of traced cattle linked to the affected herd) [16]. Although updated estimates of the costs of cage trapping have been published recently [17], in the absence of updated costs for other culling methods, or for experiencing a breakdown, we have used the 2005 estimates to ensure comparability. We based calculations on an idealised circular culling area large enough to give an overall beneficial effect over the period from completion of the first proactive cull until effects were no longer detectable. As in previous analyses [10], we assumed that only 75% of targeted land was accessible, reducing the cost of culling.

## Results

### Inside Culling Areas

**Figure 1:**
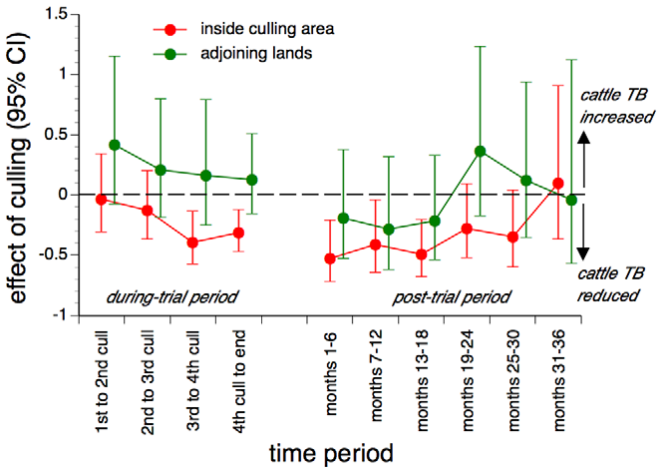
Estimated effects of proactive culling on the incidence of confirmed cattle TB breakdowns. Estimates are presented for herds inside trial areas as well as those on adjoining lands ≤2 km outside trial area boundaries. The estimated effects of proactive culling are stratified by time periods defined by the cull dates in the during-trial period, and by 6-month intervals from 1 year after the last proactive cull (the post-trial period).

Across the entire post-trial period, the incidence of confirmed breakdowns inside proactive culling areas was 37.6% lower (95% CI: 24.6% to 48.4% lower) than that inside no-culling areas (Table 1). Dividing the post-trial period into six-month intervals revealed a significant (p = 0.038) linear trend (on a log scale) over time, with the beneficial effect declining by 14.3% with each six-month interval (Figure 1). By months 31-36, no beneficial effect was detectable (Table 1). For the 30-month period when effects were detectable, proactive culling was associated with a 42.0% reduction (95% CI: 24.1-55.6% reduction) in the incidence of cattle TB.

**Table 1:** Estimated effects of proactive culling on the incidence of confirmed cattle TB breakdowns inside trial areas.

	Triplet-years	Proactive effect		Overdispersion*		P-value for linear trend over time
		Estimate (95% CI)	p-value	factor	p-value	
*During-trial period*						
1^st^ to 2^nd^ cull	12.6	−3.5% (−30.6% to 34.1%)	0.83			
2^nd^ to 3^rd^ cull	13.2	−12.8% (−36.6% to 20.1%)	0.40			
3^rd^ to 4^th^ cull	8.4	−39.4% (−57.6% to −13.4%)	0.006			
After 4^th^ cull to end	21.5	−31.5% (−46.8%to −11.9%)	0.003			
*Entire during-trial period*	55.7	−23.2% (−32.7% to −12.4%)	<0.001	0.67	0.87	0.15
*Post-trial period*						
Months 1–6	5.0	−52.7% (−71.8% to −20.8%)	0.004			
Months 7–12	5.0	−41.1% (−64.0% to −3.8%)	0.034			
Months 13–18	5.0	−49.4% (−67.9% to −20.4%)	0.003			
Months 19–24	5.0	−27.8% (−52.4% to 9.4%)	0.094			
Months 25–30	5.0	−35.0% (−59.5% to 4.3%)	0.074			
Months 31–36	3.9	9.9% (−36.7% to 90.7%)	0.74			
Months 37–42	0.4	–†				
*Entire post-trial period*	29.3	−37.6% (−48.4% to −24.6%)	<0.001	1.08	0.32	0.038
During- and post-trial periods combined	85.0	−28.7% (−35.8% to −20.8%)	<0.001	0.72	0.85	

Analyses adjust for triplet, baseline herds, and historic cattle TB incidence (over three years) and include the entire during- and post-trial periods.

*The analysis dividing both during- and post-trial periods into shorter intervals has overdispersion factor 1.21,p = 0.003; †Insufficient breakdowns to calculate estimates.

Across the combined during- and post-trial period, the incidence of confirmed breakdowns was 28.7% lower (95% CI: 20.8% to 35.8% lower) in proactive areas than in no-culling areas. For the period comprising the during-trial period and the first 30 months of the post-trial period (when beneficial effects were detectable), there was no significant linear effect of distance from the trial area boundary on the magnitude of the beneficial effect (Table 2).

**Table 2:** Estimated effects of proactive culling on the incidence of confirmed cattle TB breakdowns at varying distances inside and outside trial area boundaries, over the period from the initial culls to the end of first 30 months of the post-trial period.

	Proactive effect		Overdispersion		P-value for linear trend
	Estimate (95% CI)	p-value	factor	p-value	
*Inside trial areas*					
0–1 km inside	−20.4%(−35.4% to −2.1%)	0.031	1.39	<0.001	0.18
1–2 km inside	−25.9%(−42.8% to −4.1%)	0.022			
2–3 km inside	−31.3%(−50.3% to −5.1%)	0.023			
3–4 km inside	−22.2%(−52.8% to 28.0%)	0.32			
4–5 km inside	−46.0% (−85.7 to 103.6%)	0.36			
*Entire trial area*	−28.7%(−35.8% to −20.7%)	<0.001	0.86	0.63	
*Adjoining lands ≤2 km outside trial areas*					
0–0.5 km outside	−18.0%(−38.0% to 8.5%)	0.16	1.19	0.017	0.61
0.5–1 km outside	35.8% (2.7% to 79.5%)	0.032			
1–1.5 km outside	−2.9%(−28.1% to 31.1%)	0.85			
1.5–2 km outside	14.3%(−18.7% to 60.7%)	0.44			
*Entire area of adjoining land*	11.7%(−13.0% to 43.4%)	0.39	1.84	0.001	

Analyses adjust for triplet, baseline herds, and historic cattle TB incidence (over three years).

### Adjoining Lands

Across the entire post-trial period, the incidence of confirmed breakdowns on lands ≤2 km outside proactive culling areas was comparable (5.6% lower, 95% CI: 31.4% lower to 30.0% higher, p = 0.73) with that ≤2 km outside no-culling areas (Table 3). Dividing the post-trial period into six-month intervals revealed that the effects of culling were estimated to be beneficial for the first 18 months of the post-trial period but never significantly so (20.4% lower in the first 18 months, 95% CI: 41.3% lower to 8.0% higher, p = 0.19) (Table 3). For the 30-month period when effects were detectable inside trial areas, the incidence of confirmed breakdowns on lands ≤2 km outside proactive culling areas was comparable (6.0% lower, 95% CI: 29.7% lower to 25.7% higher, p = 0.68) with that ≤2 km outside no-culling areas.

**Table 3:** Estimated effects of proactive culling on the incidence of confirmed cattle TB breakdowns on lands ≤2 km outside trial areas.

	Triplet-years	Proactive effect		Overdispersion*		P-value for linear trend over time
		Estimate (95% CI)	p-value	factor	p-value	
*During-trial period*						
1^st^ to 2^nd^ cull	12.6	43.1%(−5.6% to 116.8%)	0.091			
2^nd^ to 3^rd^ cull	13.2	22.8%(−16.9% to 81.7%)	0.30			
3^rd^ to 4^th^ cull	8.4	17.8%(−23.4% to 81.1%)	0.45			
After 4^th^ cull to end	21.5	14.7%(−13.8% to 52.6%)	0.35			
*Entire during-trial period*	55.7	24.5%(−0.6% to 56.0%)	0.057	1.26	0.13	0.077
*Post-trial period*						
Months 1–6	5.0	−17.5%(−51.2% to 39.5%)	0.47			
Months 7–12	5.0	−26.9%(−60.0% to 33.5%)	0.31			
Months 13–18	5.0	−19.5%(−51.9% to 34.8%)	0.41			
Months 19–24	5.0	37.9%(−15.5% to 125.2%)	0.20			
Months 25–30	5.0	14.1%(−33.5% to 95.5%)	0.63			
Months 31–36	3.9	−2.1%(−55.2% to 113.8%)	0.96			
Months 37–42	0.4	–†				
*Entire post-trial period*	29.3	−5.6%(−31.4% to 30.0%)	0.73	1.51	0.025	0.17
During- and post-trial periods combined	85.0	11.7%(−12.9% to 43.2%)	0.38	1.83	0.001	

Analyses adjust for triplet, baseline herds, and historic cattle TB incidence (over three years) and include the entire during- and post-trial periods.

*The analysis dividing both during- and post-trial periods into shorter intervals has overdispersion factor 1.14, p = 0.030; †Insufficient breakdowns to calculate estimates.

Across the entire combined during- and post-trial period, the incidence of confirmed breakdowns on lands ≤2 km outside proactively culled areas was comparable (11.7% higher, 95% CI: 12.9% lower to 43.2% higher, p = 0.38) with that ≤2 km outside no-culling areas. For the period comprising the during-trial period and the first 30 months of the post-trial period (when beneficial effects were detectable inside trial areas), there was no significant linear effect of distance from the trial area boundary on the magnitude of the effect (Table 2).

### Extrapolation to Culling Areas of Different Sizes

**Figure 2:**
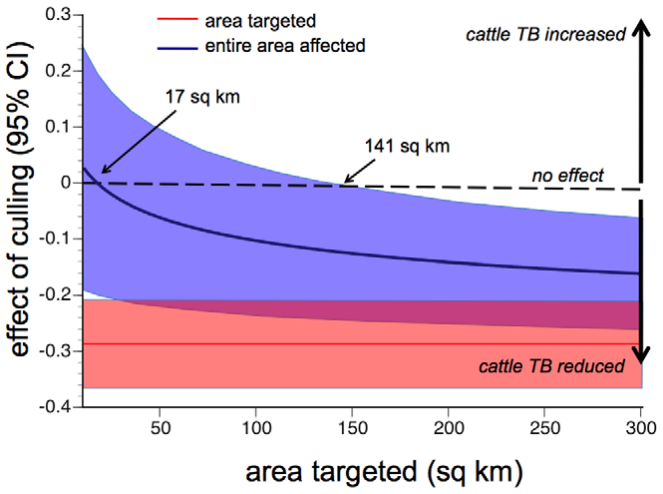
Extrapolation of overall effects to culling areas of different sizes. The blue area shows the 95% confidence interval for the overall impact (combining the impact inside the targeted area with that seen ≤2 km^2^ outside) of different sized circular culling areas. The red area shows the impact inside the targeted area only. The estimated overall effect is of increased incidence for areas smaller than 17 km^2^, moving to a decreased incidence when areas larger than 17 km^2^ are targeted. The effect of decreased overall incidence is statistically significant for areas larger than 141 km^2^.

Extrapolations to culling areas of different sizes assume an idealised circular area to be targeted by culling, surrounded by a 2 km-wide annulus of adjoining land. Since there was no significant trend in the effects by distance from the trial area boundary (Table 2), extrapolations assumed that effects were consistent throughout the affected areas. Extrapolations were based on effects over the entire during-trial period, plus the 30 months of the post-trial period when effects were still detectable. Within these assumptions, the overall average effect of proactive culling was predicted to lead to a net reduction in the overall incidence of confirmed herd breakdowns when targeted at circular areas larger than 17 km^2^ (Figure 2). However, the 95% CI for the average effect across the entire affected area only excluded net increases in the overall incidence of confirmed herd breakdowns for culling targeted at circular areas greater than 141 km^2^ (Figure 2).

### Financial Costs and Benefits

Illustrative calculations of the costs and benefits of culling covered the five-year during-trial period of annual culls (from the completion of the initial cull to one year after the fifth cull) plus the subsequent 2.5 years during which culling effects were detectable. Over these 7.5 years, in the absence of any culling, an idealised circular area of 150 km^2^, with a herd density of 1.25/km^2^ and a background incidence of 0.08 breakdowns/herd/year, would be expected to experience 112.5 herd breakdowns. Over the same period, adjoining lands (99 km^2^ falling ≤2 km outside the circular area) would experience 74.3 breakdowns, giving a combined total of 186.8. During a five-year culling period, annual proactive culling in the circular area would be expected to prevent 23.2% of 75 breakdowns inside the culled area (17.4 breakdowns prevented), while increasing the number of breakdowns on adjoining land by 24.5% (prompting 12.1 additional breakdowns), giving an overall total of 5.3 breakdowns prevented. In the 2.5 years following culling, the number of breakdowns inside the culled area would be reduced by 42.0% (15.8 breakdowns prevented), and the number on adjoining lands would be reduced by 6.0% (1.5 breakdowns prevented), giving an overall total of 17.3 breakdowns prevented. Hence, the total impact of culling such an idealised area would be to prevent 22.6 breakdowns over 7.5 years. This constitutes a saving of £610,200 at £27,000/breakdown [16]. For comparison, the cost of conducting five annual culls over a 150 km^2^ area, 75% of which was accessible for culling, is estimated as £2.14 million for cage trapping (as undertaken in the RBCT) at £3,800/km^2^/year, or £1.35 million for snaring or gassing at roughly £2,400/km^2^/year [16].

## Discussion

The results presented here show the duration of reductions in cattle TB incidence associated with widespread badger culling. Beneficial effects inside culled areas were greatest shortly after culling ended, but then declined over time and were no longer detectable four years after the last annual cull (i.e. three years into the post-trial period). On adjoining lands, the effects of culling were estimated to be beneficial only for the first 18 months of the post-trial period but never significantly so.

Although there have been a number of assessments of the effects of badger culling on cattle TB, our study provides the only experimentally-derived estimate of the duration of effects following the cessation of culling. There has been one other large-scale replicated trial of the effects of badger culling on cattle TB incidence, albeit without the randomised allocation of treatments, or the no-culling control [18]. This study, conducted in the Republic of Ireland and known as the Four Areas Trial, found reductions in cattle TB incidence ranging from 51% to 68% over a five-year culling period [18]. One explanation for the larger beneficial effect of ongoing culling observed in the Four Areas Trial is that greater reductions in badger density may have been achieved, because (i) land occupier compliance was higher; (ii) the use of snares, rather than cage traps, probably allowed a higher proportion of badgers to be captured; and (iii) the culling areas were selected to have geographical barriers such as coastline and rivers which would impede badger recolonisation. However, since culling is still ongoing in the Four Areas, that study provides no data on the duration of impacts post-culling which can be compared with the results presented here. Similarly, Kelly *et al*. [19] studied the long-term effects of badger culling on cattle TB using 16 years of observational data, but badger culling was ongoing throughout (with some periods having more intensive culling than others).

In the absence of data on badger populations during the post-trial period, we cannot be certain of the ecological and epidemiological mechanisms underlying the changes in cattle TB risks that we documented in and around former RBCT culling areas. However, we suspect that these changes reflect recovery of badger numbers and spatial organization following the cessation of culling. Proactive culling markedly reduced local densities of badgers [20], which would be expected to reduce the overall risk of cattle coming into contact with badgers. However, culling also prompted expansions of badger ranging behaviour [21], [22], increasing the number of herds that each badger could potentially contact. Moreover, culling increased the prevalence of *M. bovis* infection among badgers [23], [24]; this, combined with badgers' expanded ranging, would increase the probability of badger-to-cattle transmission, undermining the beneficial effects of reduced badger density. In another study, cessation of culling prompted a contraction of badger ranging within about two years, but recovery of badger numbers took around 10 years [25]. We previously suggested [11] that the marked reductions in cattle TB incidence observed immediately after the cessation of culling might reflect contraction of badger home ranges (and consequently reduced contact with cattle) prior to substantial recovery of badger numbers. We further speculate that the subsequent decline and disappearance of these beneficial effects may reflect increasing badger numbers, and consequently increased badger-cattle contact. While it is impossible to determine whether the system has now returned to equilibrium, in other studies badger numbers have taken five [26] to ten [25], [27] years to recover from culls, suggesting that growth of the badger populations in RBCT proactive areas may continue for several more years. As the prevalence of *M. bovis* infection in badgers was found to rise on successive culls [23], it is possible that the prevalence in badgers might still be elevated in RBCT areas (although no data are available to test this hypothesis). Were this the case, however, continued growth of the badger populations might be associated with future increases in the risk of TB transmission to cattle herds in areas proactively culled during the RBCT. Continued surveillance of cattle herds will allow characterisation of any further changes in cattle TB incidence, while studies of badger population density, spatial organization, and *M. bovis* infection prevalence could provide ecological and epidemiological insights into the long-term impacts of culling, and its cessation, on bovine TB dynamics.

It is important to note that the effects described here relate only to culling as conducted in the RBCT, *i.e.* deployment of cage traps by highly trained staff in coordinated, large-scale, simultaneous operations, repeated annually for five years and then halted. As described elsewhere, culling-induced changes in badger numbers and movement patterns mean that culling which is small-scale, patchy, short-term or asynchronous is very unlikely to provide comparable reductions in the incidence of cattle TB and could well prompt increases [8], [9], [10], [15], [21], [23], [24]. Other culling methods, such as snares or gassing, might be expected to remove a higher proportion of local badger populations than did cage traps (albeit with a likely cost in terms of badger welfare). However, since there is both ecological [20] and genetic [22] evidence that badger culling prompts substantial immigration from surrounding lands, improvements in culling efficiency might not result in proportional reductions in badger density, and would not therefore be expected to greatly improve the beneficial effects of culling. In principle, such immigration could be limited by culling within geographical features which present barriers to badger movement (as in the areas selected for culling in Ireland's Four Areas Trial [18]). However, such geographical barriers are sparse in TB-affected areas of Britain [e.g., 28]. Detailed consideration of other potential forms of badger culling [10] suggests that no practicable methods would be likely to yield benefits markedly greater than those achieved in the RBCT.

Our results suggest that culling would need to be targeted at circular areas larger than 141 km^2^ for long-term benefits to be realised. Because the relative benefits improve only slowly with increasing area culled (Figure 2), even larger areas would need to be targeted to be confident of benefits substantially greater than break-even. For example, to be confident of achieving at least a 10% reduction in the overall incidence of cattle TB would require targeting culling at circular areas ≥568 km^2^. These extrapolated figures are somewhat larger than those published most recently, because earlier extrapolations assumed that the benefits of culling increased at greater distances inside the culling area boundary [11]. Since no such trend is detectable in this updated dataset, it was excluded from the calculations presented here. All such extrapolations are illustrative: in reality, deviations from perfectly circular culling areas would increase edge effects and reduce overall benefits, while positioning of culling areas close to cattle-free areas or geographic barriers to badger movement might potentially reduce edge effects and increase net benefits [10]. Nevertheless, such extrapolations give a rough indication of the minimum areas within which culling would need to be conducted for benefits to be realised.

These updated findings also allow an assessment of the financial costs and benefits of badger culling as a tool to control cattle TB. The overall number of breakdowns estimated to be preventable by proactive culling is fairly modest in comparison with background TB incidence (e.g. 22.6 breakdowns prevented over 7.5 years in an area that would otherwise experience roughly 187 breakdowns), and the consequent financial savings much too low to offset the costs of culling using cage traps, snares, or gassing. Defra estimated that the costs of culling would be substantially lower if implemented by licencing of farmers (roughly £1,000/km^2^/year [16], hence £562,500 for the idealised five-year 150 km^2^ area described above; note that the Welsh Assembly Government recently published updated cost estimates of £4,200/km^2^/year for government-delivered cage trapping and £1,500/km^2^/year for farmer-delivered culling [17].). However, this assumed that farmers would conduct the culling themselves (and so included only minimal capital costs) and excluded the costs of training farmers or coordinating their efforts [16]. In the absence of such training and coordination, licenced culling would almost certainly be patchy, asynchronous, unsustained and uncoordinated, circumstances highly likely to prompt increases, rather than reductions, in the incidence of cattle TB [10], [15], [23], [24]. Hence, although the total cost of licenced culling is slightly lower than the potential benefits projected from RBCT results (using 2005 cost estimates [16]), it is extremely unlikely that such benefits could in fact be realised by this culling method. The costs of conducting badger culls thus substantially exceed the long-term financial benefits likely to be achieved.

Our findings are broadly consistent with those of a recent analysis [29] which assessed the potential financial outcomes of badger culling by combining a transmission model (incorporating aspects of badger ecology such as post-cull disruption of badger social organization, as well as farm management such as cattle movement) with data on costs and benefits. In this model, cage-trapping of badgers (assumed to remove 70% of badgers), produced a net economic loss in all simulations, with these losses being greater than those associated with the other culling options considered (shooting free-ranging badgers, snaring and gassing). The authors concluded “*Model results strongly indicate that although, if perturbation* [of badger social groups] *were restricted, extensive badger culling could reduce rates in cattle, overall an economic loss would be more likely than a benefit*.”

Predicting the financial implications of continuing (rather than halting) annual proactive culls is speculative. However, we can estimate the financial costs and benefits to be incurred annually in and around the idealised circular area of 150 km^2^ (with a herd density of 1.25/km^2^ and a background incidence of 0.08 breakdowns/herd/year) based on the impacts of culling estimated between the fourth proactive cull and the end of the during-trial period (the latest estimates available while the proactive culling treatment was ongoing, Tables 1 and 3). On this basis, each year of annual proactive culling in the circular area would be expected to prevent 31.5% of 15 breakdowns inside the culled area (4.7 breakdowns prevented), while increasing the number of breakdowns on adjoining land by 14.7% (prompting 1.4 additional breakdowns), giving an overall total of 3.3 breakdowns prevented on average. This constitutes an annual saving of £89,100 at £27,000/breakdown [16]. For comparison, the cost of conducting an annual culls over a 150 km^2^ area, 75% of which was accessible for culling, is estimated as £427,500 for cage trapping (as undertaken in the RBCT) at £3,800/km^2^/year, or approximately £270,000 for snaring or gassing at roughly £2,400/km^2^/year [16]. Clearly, continuing to cull would be relatively costly were the benefits of ongoing annual culling to continue at the levels observed following the fourth and subsequent proactive culls in the RBCT.

Our findings have important implications for the development of cattle TB control policies throughout the British Isles. They show that, although widespread badger culling can achieve overall reductions in the incidence of cattle TB, these benefits are not sustained in the long term once culling is halted. Moreover, the financial costs of conducting the culling substantially exceed the overall benefits accrued. In the absence of other practicable culling methods likely to yield greater benefits, our findings indicate that, on the basis of cost-effectiveness, badger culling is unlikely to contribute to the control of cattle TB in Britain.
